# High-throughput synthesis of modified Fresnel zone plate arrays via ion beam lithography

**DOI:** 10.3762/bjnano.9.194

**Published:** 2018-07-25

**Authors:** Kahraman Keskinbora, Umut Tunca Sanli, Margarita Baluktsian, Corinne Grévent, Markus Weigand, Gisela Schütz

**Affiliations:** 1 Max Planck Institute for Intelligent Systems, Heisenbergstrasse 3, 70569 Stuttgart, Germany

**Keywords:** extreme ultraviolet (EUV) radiation, focused ion beam (FIB), Fresnel zone plate, ion beam lithography (IBL), nanopatterning, soft X-rays

## Abstract

Fresnel zone plates (FZP) are diffractive photonic devices used for high-resolution imaging and lithography at short wavelengths. Their fabrication requires nano-machining capabilities with exceptional precision and strict tolerances such as those enabled by modern lithography methods. In particular, ion beam lithography (IBL) is a noteworthy method thanks to its robust direct writing/milling capability. IBL allows for rapid prototyping of high-resolution FZPs that can be used for high-resolution imaging at soft X-ray energies. Here, we discuss improvements in the process enabling us to write zones down to 15 nm in width, achieving an effective outermost zone width of 30 nm. With a 35% reduction in process time and an increase in resolution by 26% compared to our previous results, we were able to resolve 21 nm features of a test sample using the FZP. The new process conditions are then applied for fabrication of large arrays of high-resolution zone plates. Results show that relatively large areas can be decorated with nanostructured devices via IBL by using multipurpose SEM/FIB instruments with potential applications in FEL focusing, extreme UV and soft X-ray lithography and as wavefront sensing devices for beam diagnostics.

## Introduction

Requirements for focusing elements that work at extreme ultraviolet (EUV) and soft X-ray (SXR) energies are very different from those of the more familiar ultraviolet, visible or infrared regions. Virtually all matter is very absorptive in these energies, and ordinary refractive lenses do not work in this region of the electromagnetic spectrum [1]. One solution to the problem is to use specialized optics such as the Fresnel zone plates (FZPs). FZPs are diffractive lenses [2] and are often the best choice for high-resolution, high-energy beam focusing applications such as scanning transmission X-ray microscopy [3], EUV lithography (EUVL) mask inspection [4–7] and direct-write EUVL [8–9], and soft and even hard X-ray lithography [10]. When fabricated to tight tolerances, FZPs can achieve diffraction-limited focusing and imaging performance. The fabrication requirements of nanofocusing FZPs are stringent [1]. Usually, state-of-the-art electron beam lithography instrumentation is chosen to comply with these strict requirements [11–14]. Recently, a few alternative FZP fabrication techniques gained some attraction thanks to the improvements in layer deposition [15–25], etching methods [26], and fabrication methods based on focused ion beams [18,21,27–31]. One particular implementation of focused ion beams is direct-write ion beam lithography (IBL) and machining [32–34]. A well-known advantage of IBL is the ease of rapid prototyping of small-scale microfluidic, optical or electronic nanodevices. IBL has recently been applied for fabricating high-resolution functional FZPs [28,35–36] and for the successful realization of axially symmetric kinoform X-ray lenses via a gray-scale direct-write IBL approach [37].

In this work, we further demonstrate the improvements to our single-step writing of high-resolution FZPs via IBL. The means of improvements both in fabrication time and resolution by following a single-pass, single-pixel exposure (SPSP-E) writing strategy will be discussed. Then, an application of rapid realization of a high-resolution FZP with 30 nm outermost zone width and its imaging performance in a scanning transmission X-ray microscope (STXM) will be presented. Finally, the method is applied to the fabrication of an array of FZPs with similar properties and its future applications are presented.

## Results and Discussion

### Ion beam lithography

**Figure 1:**
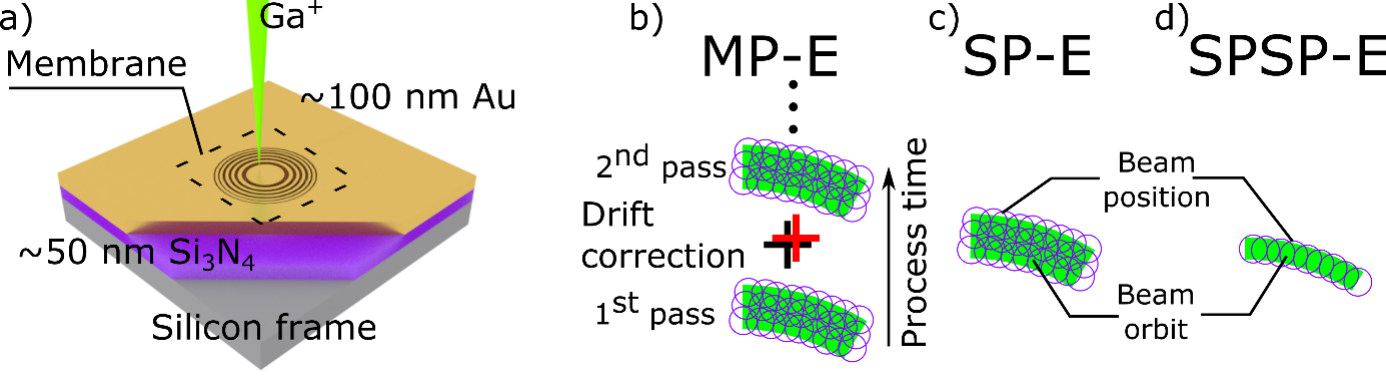
a) For the fabrication of an IBL FZP first, a ca. 100 nm gold layer is deposited on a commercial Si_3_N_4_ membrane substrate of about 50 nm thickness. Then, the FZP pattern is written in the gold film using a focused Ga^+^ ion beam. Several fabrication strategies are shown in b), c) and d). In b) a multi-pass exposure (MP-E) scheme with drift correction steps in between each cycle is followed. MP-E is a good strategy for large patterns and thicker gold films [35–36]. In c) a single-pass strategy is shown that is best for patterns with smaller features and thinner gold films [28]. Finally, in d) an SPSP-E strategy is followed where the pattern dimensions are determined solely by the beam size and the beam sample interactions. SPSP-E strategy provides a path towards fabricating smaller features.

The general fabrication route is summarized in Figure 1a and follows the deposition of a thin film lens material (Au in this case) onto an X-ray transparent substrate followed by direct-write lithography (Figure 1a). The gold thin films were deposited on commercially available amorphous silicon nitride membranes (50 nm thick Si_3_N_4_) as described in the experimental section. The ion beam lithography (IBL) was done using a scanning electron microscope (SEM), focused ion beam (FIB) dual-beam instrument, installed with a lithography attachment (Please see the Experimental section for details).

Several exposure, milling or patterning strategies can be adopted in an IBL process. A few such processing procedures relevant to present work are illustrated in Figure 1b–d. Removal of large volumes of material (for instance, 100 µm diameter, 500 nm gold thickness [36]) usually means lengthy processes that require an multi-pass-exposure (MP-E) strategy as depicted in Figure 1b, and involves drift correction steps in between cycles [29,35–36]. In some cases, the drift correction can be unnecessary, but the MP-E can still be desired when a better dose distribution or a well-defined wall geometry is aimed for in structures with higher aspect ratio [38]. We have shown that a much faster process can be devised by employing a single-pass-exposure (SP-E, Figure 1c) strategy for FZPs with smaller dimensions (50 µm diameter and 100 nm thickness). In a previous work, the SP-E method enabled fabrication of higher-resolution (50 nm Δ*r*) dense structures [28], as there is a sputter enhancement in SP-E [39] due to the rapidly changing geometry of the target under the ion bombardment [40–41].

Here, we follow a slightly different strategy that provides significantly higher resolution. The approach uses a single-pixel-single-pass exposure (SPSP-E) strategy for defining the positions of the open zones. In the SPSP-E strategy, (Figure 1d), it is possible to reach even higher structural density with an effective Δ*r* down to 30 nm, without compromising the diameter and the thickness of the FZP which were 50 µm and 100 nm, respectively (Table 1).

**Table 1:** Overview of the FZP and ion beam lithography process parameters.^a^

FZP	Material	*D* (µm)	Δ*r* (nm)	*t* (nm)	δ_Rayleigh/2_ (nm)	DE@1.2 keV (%)
M-IV^*^	Au	50	30	100	18.3	7.81 (4.95)

FIB	*V* (kV)	*I* (pA)	*d* (nm)	strategy	step size (nm)	pixel dwell time (ms)
Ga^+^	30	30	16	SPSP-E	8	0.2133

^a^*D*: FZP aperture, Δ*r*: outermost zone width, *t*: nominal thickness, δ_Rayleigh/2_: expected half-pitch Rayleigh resolution, DE@1.2 keV: the diffraction efficiency for a line-to-space ratio of 1:1 according to thin grating approximation (TGA) and in parenthesis the DE of zones with L:S = 2.5:1 according to the coupled wave theory (CWT), *V*: acceleration voltage, *I*: beam current, *d*: nominal spot size. ^*^Internal sample designation.

In the SPSP-E strategy, a single pixel line is positioned on the zone centers, and the beam will scan the path only once and there will be no adjacent passes as opposed to MP-E or SP-E milling strategies. Therefore, the size of the feature to be written is defined by the ion beam spot size, the interaction volume of the ions within the material and the extent of the collateral damage of the beam tails and secondary sputtering processes. The idea here is, if the desired depth of an open zone can be reached before destroying the adjacent zones, it becomes possible to write very dense structures, very quickly. To achieve this goal, the ion beam dosage, which is now determined by the 1D beam overlap (i.e., the step size in the beam path), the current and the dwell time need to be precisely adjusted.

Following the structuring of the zones, a ca. 3 µm thick beamstop was deposited in the central inactive region via focused ion beam induced deposition (FIBID) of Pt using trimethyl(cyclopentadienyl)platinum(IV), (CH_3_)_3_CH_3_C_5_H_4_Pt, as the metal-organic precursor gas.

### Structure of the FZP

**Figure 2:**
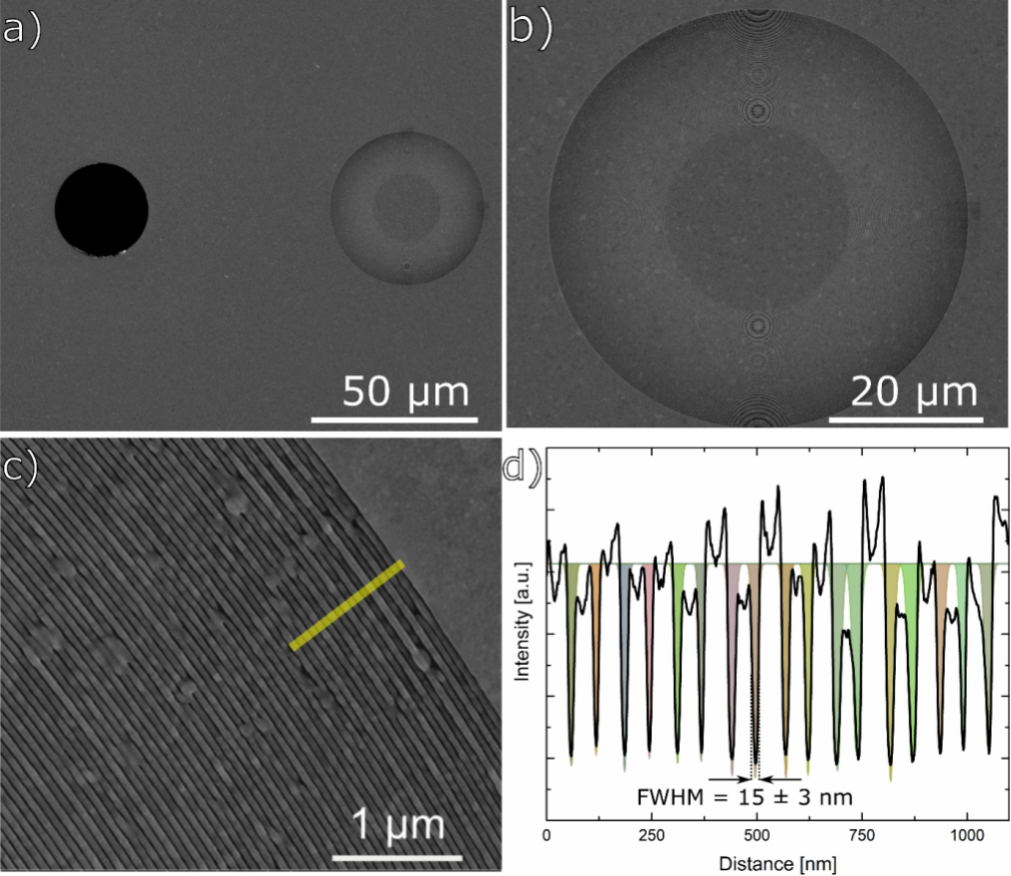
SEM images of M-IV IBL-FZP prior to the beamstop deposition. a) An overview image. The FZP and the reference aperture are shown side by side. b) A higher-magnification image showing the empty central portion and the active zones of the FZP. The circular structures over the zones are due to a moiré effect during imaging. c) Top-right part of the FZP. 60 nm wide periods can be seen. d) The line profile taken from a region roughly 30 pixels wide marked in c. The lines written with the ion beam are fitted with a Gaussian curve with an average FWHM of 15 ± 3 nm. The measured line-to-space ratio for the outermost period is roughly 2.5:1 (ca. 43:17 nm). All SEM images were recorded under normal incidence.

The patterning and ion beam parameters tabulated in Table 1 resulted in a linear dosage of 0.8 pC/µm, and the successful fabrication of the FZP with 50 µm diameter, 110 nm nominal thickness and 30 nm Δ*r* in just 8 min and 23 s. The dosage is given in linear terms because of the single pixel circular elements used for patterning. The overall FZP exhibited a high quality as shown in Figure 2. The FZP (internally designated as M-IV), is shown side by side with the reference aperture for diffraction efficiency (DE) measurements in Figure 2a. With 50 µm diameter and 30 nm ∆*r* the FZP has the same light collection capability as our previously reported high-resolution FZP [28] while having 40% smaller features. With a fabrication time of 8 min 23 s, the process was also significantly faster than that we reported previously with a reduction of 35% in total time. Thanks to the speed of fabrication, the maximum shift of the central zones caused by drift during the process were estimated to be less than 100 nm.

Due to the particular nature of the writing strategy, the line-to-space ratio (L:S) continuously decreases towards the outermost zones of the FZP from ca. 8:1 in the innermost zone, while the local grating period decreases towards the peripheral part according to the zone plate law. The high L:S means less light is transmitted through the FZP compared to an un-modified FZP. The SPSP-E milling strategy affects the FZP efficiency as discussed below. To remedy the effects of the high L:S on the efficiency, an SP-E milling strategy may be employed for the inner zones. However, this would undoubtedly increase the total process time required for completing the structure.

The quality of the outermost zones was markedly high, demonstrating the reliability of the SPSP-E process. On average, a 60 nm period was successfully achieved in the outermost part, with consistent quality around the FZP as shown in the SEM images of Figure 2b,c. The line profile over the last 17 periods, taken from the marked region in SEM image of Figure 2c is plotted in Figure 2d, and the transmitting zones written by the ion beam were fit with Gaussian profiles. The FWHM of the Gaussian fits were 15 nm with a standard deviation of 3 nm. The resulting line-to-space (L:S) ratio in the outermost part was measured to be ca. 2.5:1. Despite being able to write open-zones with a width of 15 nm (± 3 nm standard deviation), due to the 60 nm outermost period, an effective ∆*r* of 30 nm was achieved, defining the spatial resolution. These results show that there is room for further improvement in decreasing the period and hence increasing the resolution of the optic.

Figure 2b and Figure 2c show some hard Au grains remained relatively unharmed by the ion beam due to the strong dependence of the ion beam damage on the crystal orientation concerning anisotropic sputter yield and channeling effects [42]. These grains have a random spatial distribution, which renders them tolerable regarding imaging performance, though they may be expected to reduce the total transmitted light.

With a critical length of 60 nm of the outermost period and an effective ∆*r* of 30 nm, the FZP design was a challenging task for direct-write Ga^+^ ion beam lithography. Its successful realization demonstrates the capabilities of modern focused ion beam instrumentation for direct-write lithography.

### Soft X-ray microscopy tests using the FZP

**Figure 3:**
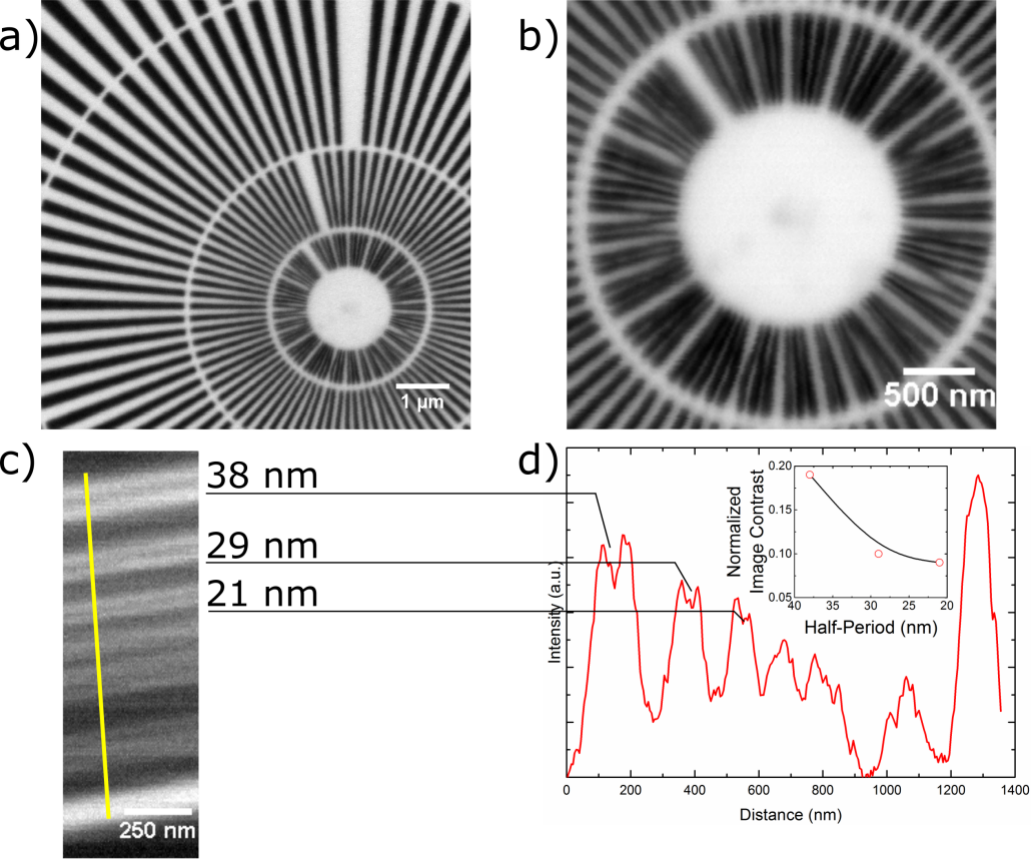
a) Soft X-ray image of the Siemens star recorded at 1 keV with 0.94 ms pixel dwell time and 11 nm step size. b) A higher-magnification image of the central ring recorded using 10 nm steps size and 10 nm dwell time. It can be seen that the smallest 30 nm features of the Siemens star are clearly resolved. c) An STXM image of the multilayer test object recorded at 1.12 keV with 30 ms dwell time and 5 nm step size. The 21 nm half-pitch features are resolved and the intensity profile in d), where the inset shows the normalized contrast of the first three features in the profile.

The imaging resolution and the DE of the FZP were tested using a scanning transmission X-ray microscope (STXM) [43] as described earlier [28]. The resolution of the FZP was tested using a Siemens star test object with features down to 30 nm and a standard multilayer test object made out of GaAs/Al_0.7_Ga_0.3_As with features down to 3 nm [17]. Figure 3a, recorded at 1 keV X-ray energy, shows that all features of Siemens star are resolved in all directions with equally high contrast. The innermost spokes were resolved by a line-by-line scan, using a relatively short pixel dwell time of 0.94 ms. A higher-magnification image of the innermost portion was obtained by a point by point scan shown in Figure 3b. It is seen from this image that the 30 nm features were resolved in both *x*- and *y*-direction with high sharpness and contrast, revealing the defects in the test object coming from its fabrication process.

In order to test the ultimate resolution of the optic, the certified calibration standard BAM L-200 sample was imaged at 1.12 eV. The raw gray-scale image in Figure 3c exhibits the 21 nm wide features, which are resolved. Further analysis of the line profile taken from Figure 3c shows significant contrast for the 21 nm feature size (Figure 3d and its inset) as demonstrated by the normalized image contrast [17] value calculated from the profile plot. The achieved resolution of (21 nm half-pitch) is remarkable considering the FZP was manufactured just in 8 min 23 s. Furthermore, it has the same aperture as the previously reported IBL-FZP [28], even though it has approximately 40% smaller features and 35 % shorter fabrication time.

The DE of the device was experimentally determined as a function of the incoming photon energy. The maximum measured DE of the FZP was measured to be 0.60% at 700 eV and decreases to less than 0.45% at 1200 eV. The DE of the whole device including the silicon nitride membrane starts to decrease for energies below 800 eV as the absorption in the underlying S_3_N_4_ layer increases. The relatively low DE can be attributed to several sources. The first factor is the significant deviation of the line-to-space ratio (L:S) from 1:1 due to the SPSP-E fabrication process (Supporting Information File 1, Figure S1). The effects of the L:S ratio on the DE at 1200 eV was calculated by using coupled wave theory (CWT) [44] and is shown in Supporting Information File 1, Figure S2. According to the CWT, the L:S ratio of 2.5:1 (outermost zones) has about 4.95% DE at 1st diffraction order as opposed to an equivalent FZP with 1:1 lines (7.8%). An L:S of 8:1 even further suppresses the DE at 1st order focus to 1.06%. As the gold lines are thicker than the spaces, some of the light is directly absorbed in the gold zones leading to lower efficiency values.

Moreover, CWT calculations demonstrate that some of the incident photons are redirected to higher orders including the 2nd and 3rd diffraction orders. 2nd order DE, which is almost 0 for a perfect 1:1 duty cycle (Supporting Information File 1, Figure S2), increases if the L:S ratio deviates from 1:1. The situation can be remedied relatively easily by switching to an SP-E process for the zones that are broader than 50 nm, at the cost of increasing the fabrication time.

The second reason for relatively low efficiency is that the outermost zones usually have smaller thicknesses than the nominal thickness value, e.g., 50 nm vs 110nm, as demonstrated earlier [28]. As shown as a shaded region in Figure S2 of Supporting Information File 1, the variations in both thickness and L:S result in a range of possible diffraction efficiencies that the zones can exhibit from innermost to outermost part of the zone plate. As the lower thicknesses lead to lower DEs in this case, the 7.8% DE given in Table 1, calculated using TGA for the nominal film thickness grossly overestimates the theoretical diffraction efficiency of the actual fabricated device.

The third and a critical factor contributing to the strongly suppressed DE is the parasitic Pt deposition during the fabrication of the beamstop via FIBID. The parasitic deposition layer, where a Pt/C layer deposits unintentionally on the regions adjacent to the actual region of interest, covers the zones with a thin layer of platinum/gallium/carbon mixture (see Supporting Information File 1, Figure S3), which absorbs incident X-rays and leads to a decrease in light transmission and hence, the efficiency. The extent of the parasitic deposition and its impact on the DE is discussed in more detail elsewhere [45].

The imaging resolution of the FZP with a variable L:S fabricated by an SPSP-E process does not differ from that of a standard FZP of the same outermost period as also proven by imaging simulations shown in Supporting Information File 1, Figure S4.

### Fabrication of the FZP arrays

**Figure 4:**
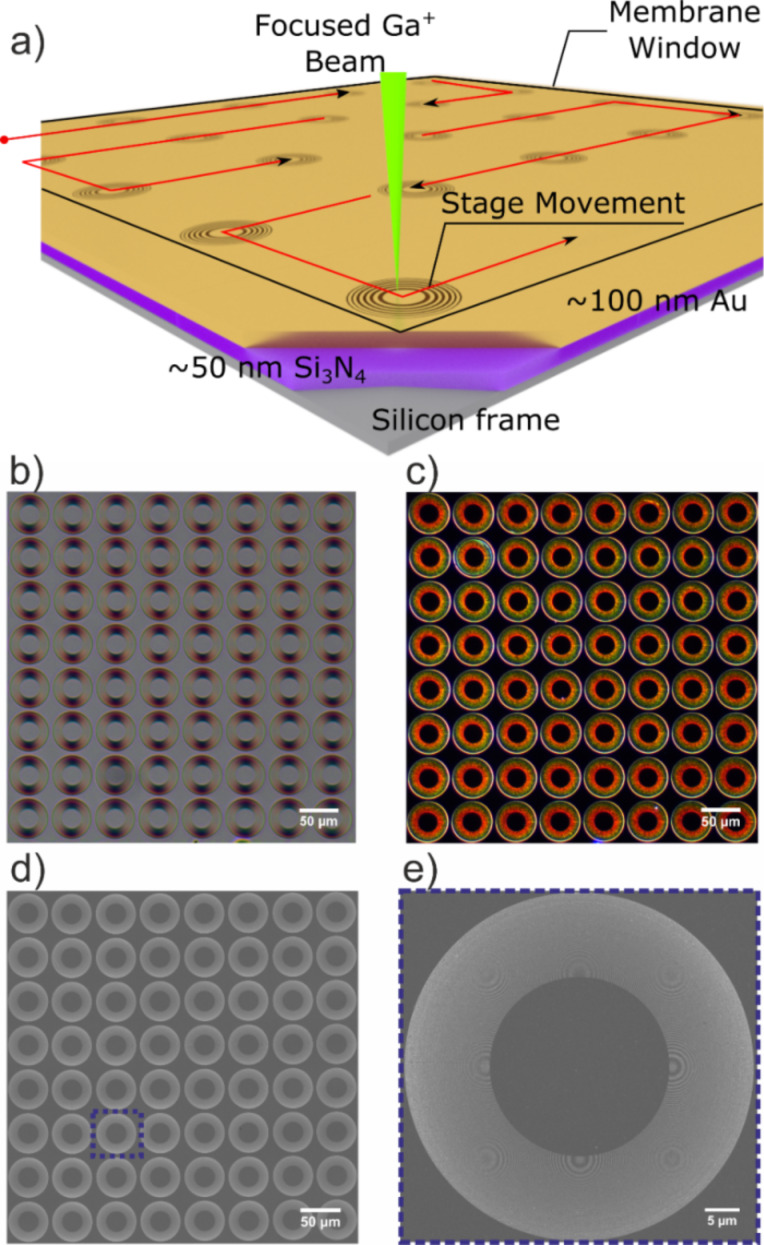
a) The fabrication scheme for an array of FZPs. The beam is scanned over the region of interest to write the FZP pattern, then, the stage moves to the new FZP position, and the process is repeated. b) Bright-field optical microscopy image of the array under polarized light. The familiar cross-shaped reflected light from the FZPs is an indicator of high quality. c) Dark-field optical image without the polarizer. The blue-shifted reflected intensity from inner zones to outer zones is attributed to shifting plasmon resonances of the zones made out of gold. d) A STEM-DF image of the fabricated 8 × 8 array of 64 FZPs. e) A STEM-DF image of FZP of row 6 and column 6.

The process described so far was employed in fabrication of an 8 × 8 matrix of FZPs on a gold-coated silicon nitride window that is 500 × 500 µm^2^ wide as illustrated schematically in Figure 4a. A single FZP was written using the parameters described above, and then the stage was driven to the next FZP position until all 64 FZPs were finished in an automated overnight process. The writing of 64 FZPs using the given parameters takes less than 10 h including the stage travel and calculation overhead.

The cross-shaped reflected intensity pattern seen in all the FZPs in Figure 4b is a polarized bright-field optical microscope image and a sign of overall high quality [46]. The cross shape rotates as the polarization is varied between 0° and 90°. The dark-field image of the array reveals a color change as a function of zone pitch. In the center, where the zone period increases up to 115 nm, the reflected intensity is brightest in red. As the period decreases towards the outer zones down to 60 nm, the reflected intensity shifts to green and then to blue. This can be attributed to the shifting plasmon resonances of the gold nanowires that make up the zones of the FZPs.

A scanning transmission electron microscope (STEM) dark-field overview image of the completed array is shown in Figure 4b. The FZPs mostly exhibited high quality with zones free from defects. Only in three of the FZPs minor defects in a few zones were observed, which is expected to reduce the DE slightly but does not hinder the function of the optic overall. The flaws were attributed to the local variations in the film structure and stress. The majority of the FZPs was intact and could pass the inspection via electron microscopy. Hence, the robustness of the fabrication method is supported with a 100% yield. One of these FZPs from the marked region in Figure 4d is depicted in Figure 4e, a higher-magnification STEM dark-field image. The FZP showed similar characteristics to the FZP structure shown in Figure 2, as expected. These arrays can be useful in applications such as zone plate array lithography [8–9], one shot X-FEL focusing or in combination with a matching array of order-sorting apertures, they can be used to construct a Shack–Hartmann [47] wavefront sensors for beamline diagnosis applications.

## Conclusion

High-resolution ion beam lithography of structures with critical dimensions down to 60 nm period was realized in gold-coated silicon nitride membranes. The fabrication time of a single FZP was 8 min 23 s. The fast fabrication scheme was achieved by exposing the zones using single-pixel lines in a single-pass milling strategy writing zones as small as 15 ± 3 nm in width. With an outermost L:S ratio of roughly 2.5:1, an effective ∆*r* of 30 nm was achieved. The FZP was tested by using the optic directly as a focusing optic in a scanning transmission X-ray microscope, resolving features of 21 ± 0.65 nm in width. While the process allows for the fabrication of quality zones, the high L:S ratio inherent to the SPSP-E process and parasitic Pt deposition during FIBID of beamstop led to suppressed efficiencies down to 0.6%. Finally, the optimized approach was applied to the fabrication of a large array of 64 FZPs in an overnight process. Such arrays of FZPs are proposed as expendable, cheap and high-resolution FZPs for FEL experiments, for lithography applications or wavefront sensing and beam diagnostic applications.

## Experimental

### Fabrication and SEM/STEM characterization of the FZPs

The 100 nm gold films were sputtered using a Leica EM ACE600 on 50 nm thick commercial Si_3_N_4_ membranes (Silson), without any rotation or tilt. The FZPs were fabricated using a Nova Nanolab600 (FEI) attached with an Elphy Multibeam (Raith) pattern generator. A 30 keV, 30 pA Ga^+^ focused ion beam with a nominal beam size of 16 nm was utilized. By using a step size of 8 nm and a dwell time of 0.2133 the linear dosage was 0.8 pC/µm. The array was fabricated by replicating the pattern in a matrix form with 55 µm steps in the *x*- and *y*-directions. Dark-field scanning transmission electron microscope images were taken using the STEM mode of the Nanolab600.

### Scanning transmission X-ray microscopy experiments

FZPs were mounted as the focusing optic in a state-of-the-art STXM, MAXYMUS [43], located at UE46-PGM-2 beamline of BESSY II facility in Berlin, as described before [28]. An energy range from 400 to 1600 eV is routinely used in this microscope in which we also have tested out FZP. Two test objects, a Siemens star (ZEISS) with 30 nm smallest features and a multilayer test sample made out of GaAs/Al_0.7_Ga_0.3_As (BAM L-200) were used to determine the resolution. The efficiency was measured by scanning a pinhole of known dimensions over the FZP and the reference hole at each energy as described earlier [18,28].

## Supporting Information

Additional SEM images of inner and outermost zones, CWT calculations about how the L:S affects the DE, SEM images of zones before and after the Pt deposition, and imaging simulations for modified and non-modified FZPs.

File 1 Additional experimental data.
