# Supplementary material for: High-throughput synthesis of modified Fresnel zone plate arrays via ion beam lithography
Source: Beilstein J Nanotechnol. 2018 Jul 25;9:2049–56. doi: 10.3762/bjnano.9.194 (PMC6071703; doi:10.3762/bjnano.9.194)
Supplement: File 1 — Additional experimental data. [file Beilstein_J_Nanotechnol-09-2049-s001.pdf]

Supporting Information

for

High-throughput synthesis of modified Fresnel zone plate arrays via ion beam lithography

Kahraman Keskinbora*, Umut Tunca Sanli, Margarita Baluktsian, Corinne Grévent, Markus Weigand and Gisela Schütz

Address: Max Planck Institute for Intelligent Systems, Heisenbergstrasse 3, 70569 Stuttgart, Germany

Email: Kahraman Keskinbora* - keskinbora@is.mpg.de

* Corresponding author

Additional experimental data

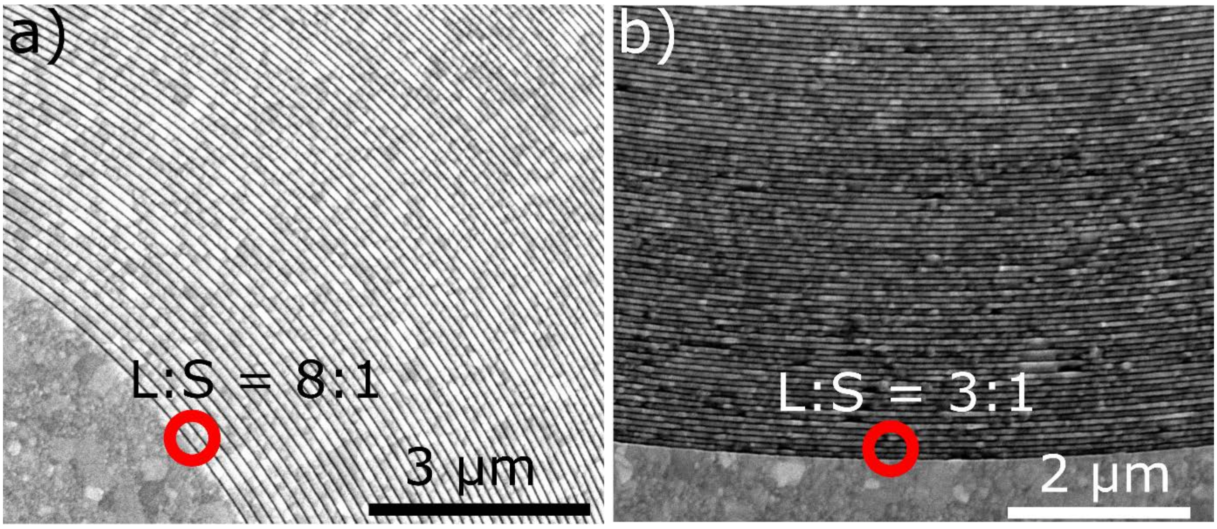

Figure S1: Effects of a pure single pixel exposure (SPSP-E) process on the L:S ratio of the FZP. a) SEM image shows the inner part of the FZP with approximately 8:1 L:S ratio. b) SEM image of the bottom part of the FZP shows an L:S of about 2.5:1 to 3:1.

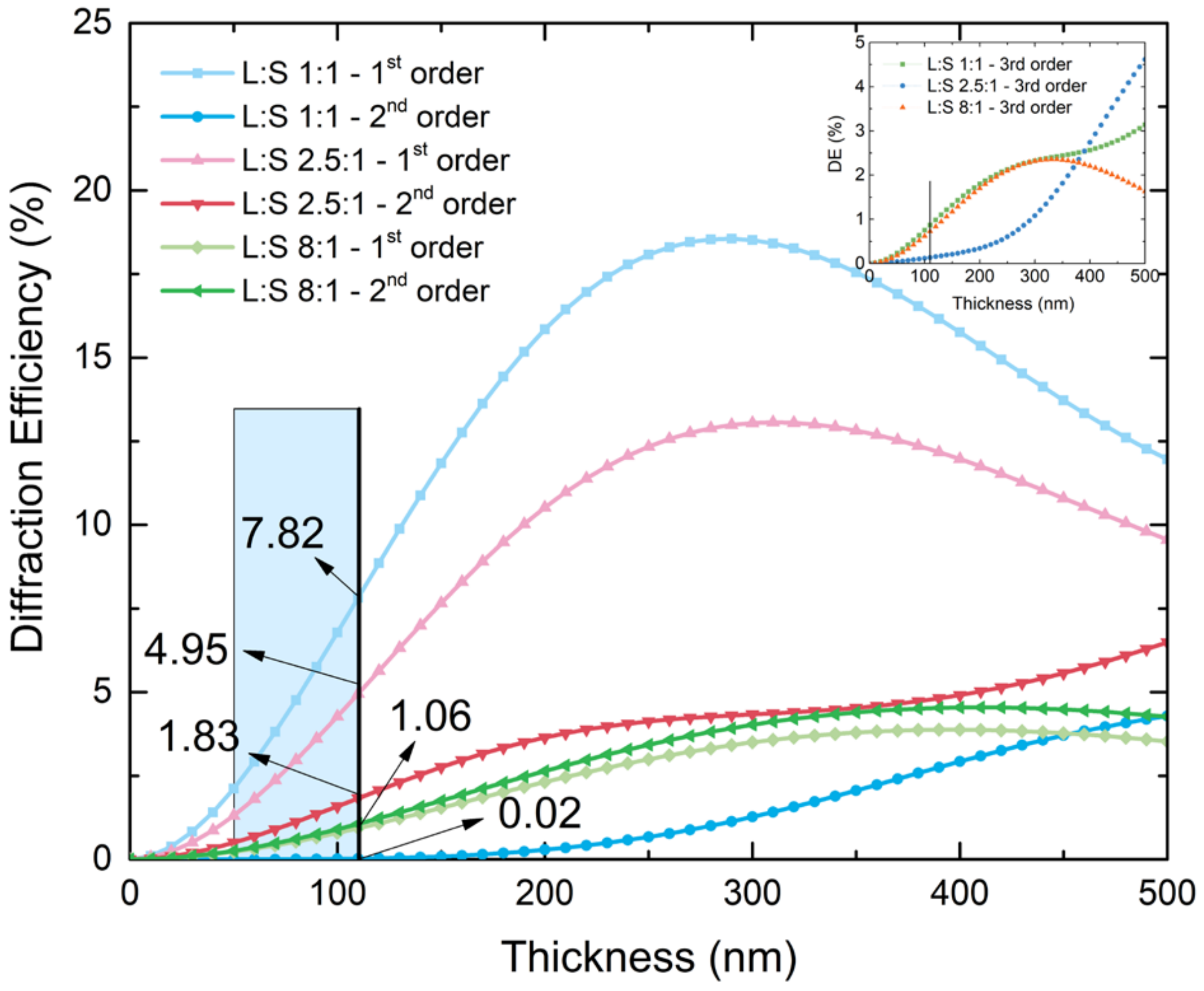

Figure S2: Effect of L:S ratio on the 1st, 2nd and 3rd order diffraction efficiencies according to the coupled wave theory at 1200 eV. The labeled efficiency values are given for a nominal zone thickness of 110 nm. At this thickness, the ratio of the intensity delivered to the 2nd order focus increases as L:S increases, and for innermost zones 1st and 2nd order focus receive almost the same energy. Outermost zones contribute more to 1st order in comparison to the inner zones, but as expected this is still below that of an ideal FZP. The shaded region corresponds to the range of theoretical efficiencies that can be expected for the FZP described here. The general trend is innermost large structures are closer to 110 nm nominal thickness while outermost zone height decreases due to secondary sputtering effects. 3rd order DE (inset) is expected to be below 1% for all cases considered here.

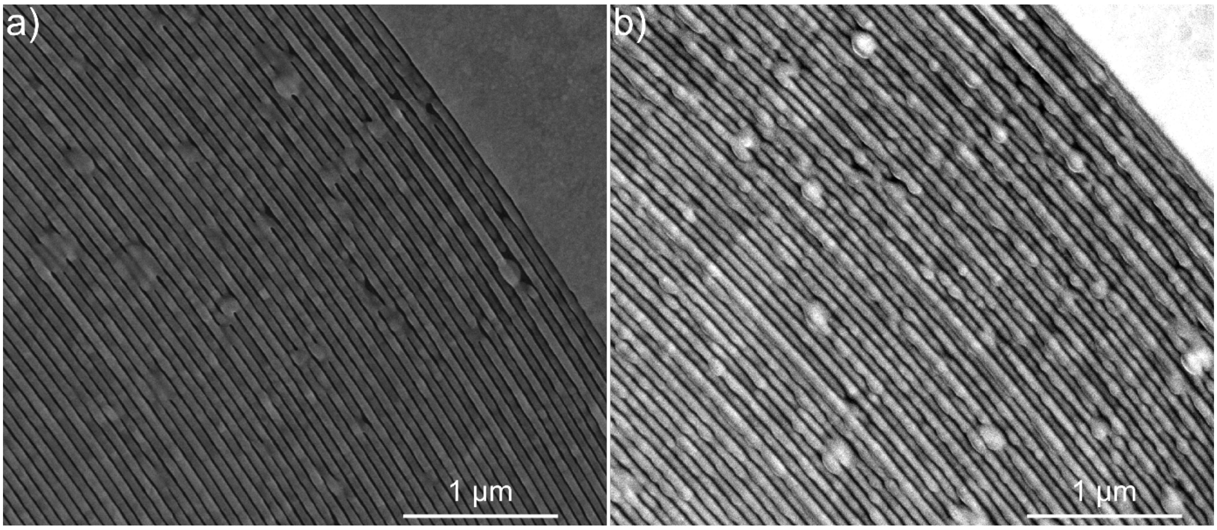

Figure S3: The effect of the parasitic platinum deposition during deposition of the beamstop via FIBID. The SEM images demonstrate the outermost region, a) before the Pt deposition and b) after the Pt deposition process.

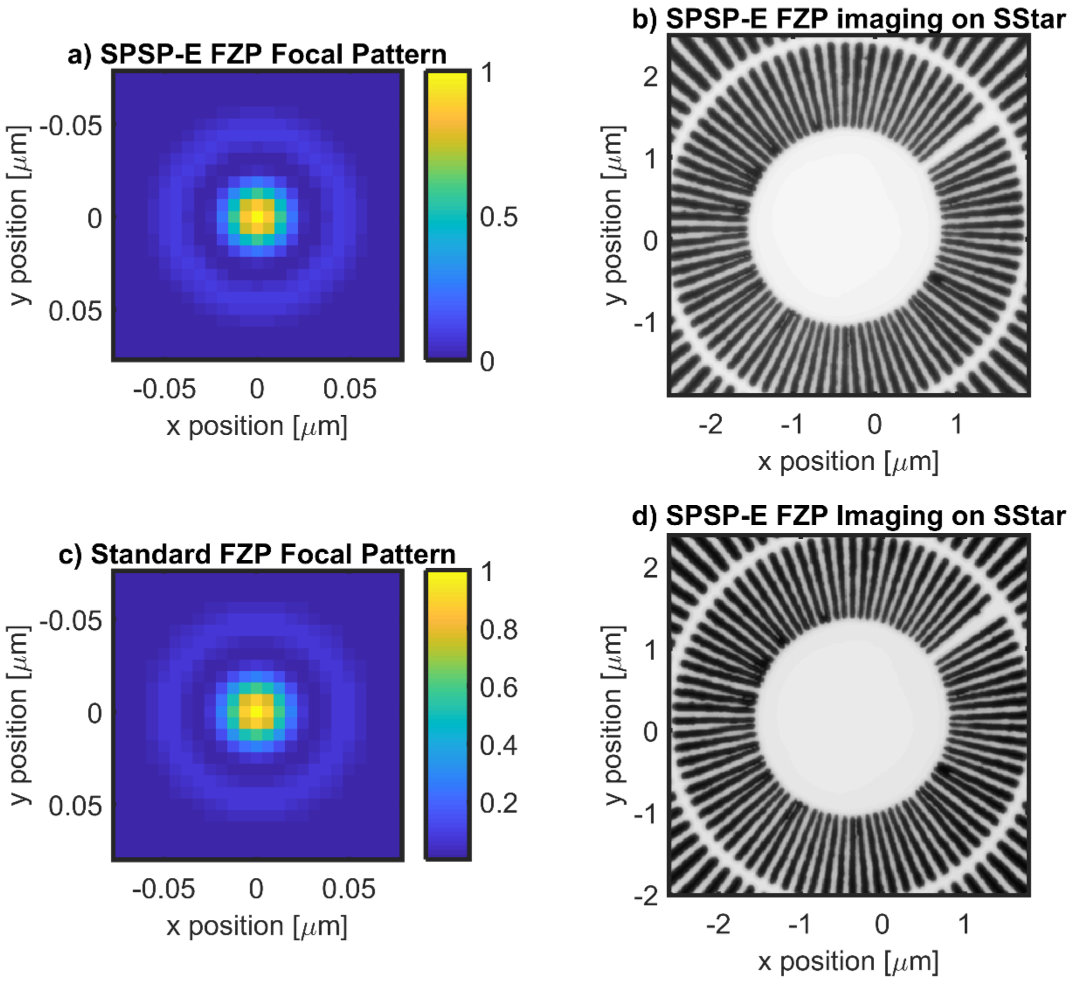

Figure S4: Comparison of the focal plane pattern (a) and simulated STXM image of the Siemens star (b) for an FZP with a variable L:S as described in the main text, to those of a standard FZP (c and d).
